# Morphological and vascular evidence of glaucomatous damage in myopic guinea pigs with scleral crosslinking

**DOI:** 10.1038/s41598-023-48461-2

**Published:** 2024-01-02

**Authors:** Lei Guo, Jun Tao, Ziqi Guo, Yang Tong, Shichao Chen, Xin Zhao, Rui Hua

**Affiliations:** 1 Ophthalmology and Optometry Centre, The First Hospital of China Medical University, Shenyang, China; 2 Department of Ophthalmology, Shenyang Sinqi Eye Hospital, Shenyang, China; 3 The First Clinical College of Jinzhou Medical University, Jinzhou, China; 4 Department of Ophthalmology, The First Hospital of China Medical University, No. 155, Nanjingbei Street, Heping District, Shenyang, 110001 Liaoning People’s Republic of China

**Keywords:** Refractive errors, Eye manifestations

## Abstract

Guinea pigs are often used as models for myopia studies. However, the imaging structure and vasculature of the optic nerve head (ONH) in guinea pigs are tentative. This study investigated morphological parameters and vascular characteristics of the ONH in guinea pigs with form deprivation (FD) myopia before and after scleral crosslinking (CXL), using optical coherence tomography (OCT) and OCT angiography (OCTA). Refractive error, axial length (AL), intraocular pressure (IOP), and OCT-based structural parameters of the ONH were measured at baseline and 3 weeks after the FD + CXL procedure in guinea pigs. The 88 guinea pigs analysed in this study were aged 3 (n = 29), 4 (n = 51), and 5 (n = 8) weeks. The IOP, AL, average and vertical cup-to-disc ratio (C/D), circumpapillary retinal nerve fibre layer, disc area, and cup volume increased at 3 weeks compared to baseline values (all p < 0.001). The refractive error and rim area decreased at 3 weeks compared to baseline values (all p < 0.001). After adjustment for age, IOP was correlated positively with average C/D (p = 0.039) and negatively with rim area (p = 0.009). The severity of blood signal defects was positively associated with the average C/D at 3 weeks (p = 0.027). These findings may facilitate further research on myopia using guinea pigs.

## Introduction

In addition to glaucoma, guinea pigs have been increasingly used as models of myopia. In the pathogenesis of myopia, the optic nerve head (ONH) is an important structure^1^. The guinea pig ONH is a histologically well-organized, collagen-based lamina cribrosa (LC) that is more similar to the human LC than to the collagen-less ONH of other rodents^2^. However, the imaging structure and vasculature of the ONH in guinea pigs have been rarely reported.

Optical coherence tomography (OCT) and OCT angiography (OCTA) have been used to qualitatively and quantitatively investigate the fundus of guinea pigs, respectively. In vivo morphometric imaging of the retina and choroid in guinea pigs is based on OCT, with acceptable inter-observer reproducibility and intra-observer repeatability^3^. Using in vivo OCT imaging, Jnawali et al.^4^ measured the thicknesses of the choroid, outer retina, ganglion cell/nerve fibre layer, and retina to be 64.8 ± 11.6 μm, 72.4 ± 2.4 μm, 59.2 ± 4.5 μm, and 147.7 ± 5.8 μm, respectively, with a Bruch membrane opening area of 0.192 ± 0.023 mm^2^. However, as a new non-invasive imaging modality without dye, OCTA assesses the ONH vasculature qualitatively and quantitatively. Specifically, this method correlates topography with the functional abnormalities observed in the visual field examination and structural abnormalities identified on OCT^5^.

Zhou et al., using OCTA, reported that scleral hypoxia is attenuated by increased choroidal blood perfusion in guinea pigs, inhibiting the development of myopia^6^. Vessel density reduction on OCTA can aid in monitoring the progression of advanced glaucoma^5^. Myopia progression is associated with a change in intraocular pressure (IOP). Young Chinese people with high myopia tend to have greater IOP with a thicker central cornea^7^. Lowering IOP inhibits axial length (AL) elongation in pathological myopia by inhibiting scleral fibroblast activation, decreasing the scleral distending force to reduce scleral remodelling, and increasing choroidal blood perfusion to reduce scleral hypoxia. However, no study has examined the relationship between the structural and vascular features of the ONH of guinea pigs under high IOP.

Our group was the first to report the vascular and morphological parameters of the ONH in normal guinea pigs at 3–4 weeks of age using in vivo OCT and OCTA imaging for qualitative and quantitative assessments, respectively^8^. Inhibiting myopic progression and axial elongation in form deprivation (FD) increased IOP and subsequent optic disc, anterior segment, and scleral changes induced by scleral crosslinking (CXL) of the guinea pig’s eyes^9^. We investigated vascular and morphological characteristics of the ONH of guinea pigs with FD myopia using OCT and OCTA before and after the CXL procedure to assess the relationship between ONH blood flow signal patterns and its structural parameters and ONH ischemia and high IOP.

## Methods

### Animal model establishment

We selected the right eyes of 200‒240 g tricolour guinea pigs without any ocular disease. The animal handling and experimentation in the study adhered to the ARVO Statement for the Use of Animals in Ophthalmic and Vision Research. All experimental protocols were approved by the Animal Care and Use Committee of the China Medical University. This study followed the ARRIVE guidelines. All methods were performed in accordance with the relevant guidelines and regulations. All guinea pigs underwent an FD + CXL procedure, as described previously^8^. We used 29-gauge needles and 0.10 ml of 0.50% genipin (FUJIFILM Wako Pure Chemical Industries, Osaka, Japan) for the subtenon injection, administered 3.0 mm behind the corneal limbus in the superotemporal and inferonasal quadrants of the studied eyes. As a routine surgical procedure with postoperative antibiotics, the study did not have humane endpoints.

### In vivo measurements

Following a previous study^8^, we measured the following parameters at baseline and 3 weeks after the FD + CXL procedure: refractive error (streak retinoscope, YZ-24, Suzhou Liuliu Vision Technology; Suzhou, China); IOP (TONOLAB tonometer, Tiolat; Helsinki, Finland); ocular AL (the manual mode of A-mode ultrasound, Aviso, Quantel Medical Inc., France); and the optic disc (spectral-domain OCT, SD-OCT, Cirrus HD 5000, Zeiss; Germany) and its OCTA function under general anaesthesia by intraperitoneal injection of 1% luciferin sodium. The analyzed parameters included the circumpapillary retinal nerve fibre layer (cpRNFL, μm) thickness, average cup-to-disc (C/D) ratio, vertical C/D ratio, disc area (mm^2^), rim area (mm^2^), and cup volume (mm^3^). OCT and OCTA measurements were automated using built-in software.

OCTA and its parameters have been applied widely for assessing ONH ischemia. For example, during deterioration in diabetic retinopathy, microvascular defects in the ONH may occur earlier than cpRNFL thinning^10^. Moreover, glaucomatous eyes have a significantly lower thickness of cpRNFL and vascular density of ONH compared with normal control eyes^11^. This study is the first to investigate the blood flow signal patterns of the guinea pig ONH to assess ONH ischemia under high IOP.

**Figure 1:**
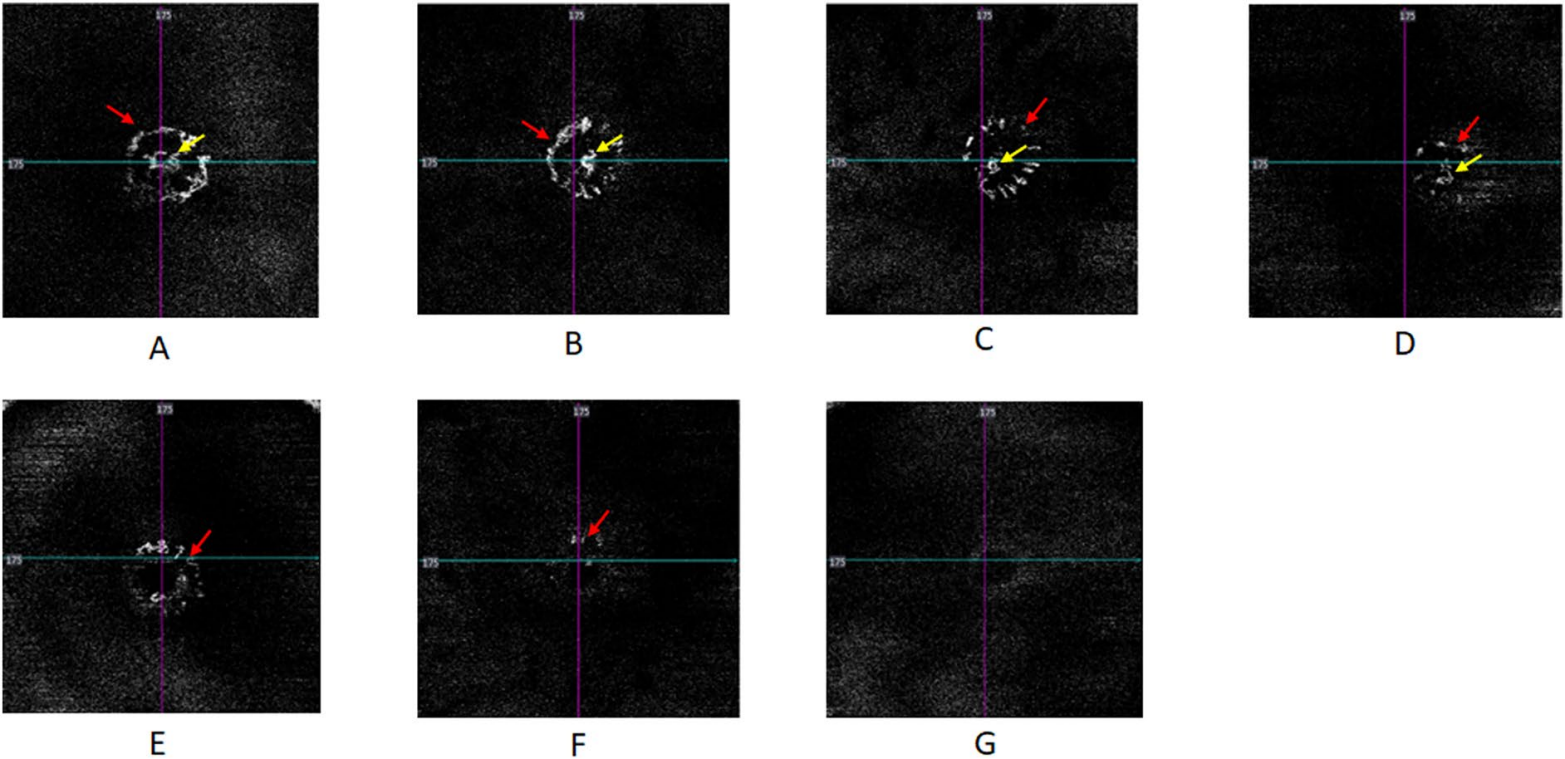
Blood flow signal patterns within the ONH regions on OCTA. (**A**) Findings for guinea pig #10 at baseline: Level one (normal blood flow signal) shows a central peak blood signal (yellow arrow) and intact peripheral vascular ring (red arrow). (**B**) Findings for guinea pig #10 at 3 weeks: Level one (normal blood flow signal) shows a central peak blood signal (yellow arrow) and an intact peripheral vascular ring (red arrow). **(C**) Findings for guinea pig #18 at 3 weeks: level two shows a peripheral vascular ring defect of < 1/3rd (red arrow) but with a central peak blood signal (yellow arrow). (**D**) Findings for guinea pig #8 at 3 weeks: level three shows a peripheral vascular ring defect of < 2/3rd (red arrow) with a normal central peak blood signal (yellow arrow). (**E**) Findings for guinea pig #2 at 3 weeks: Level three does not show a central peak blood signal with a peripheral vascular ring left (red arrow). (**F**) Findings for guinea pig #12 at 3 weeks: level four shows a blood flow signal defect > 2/3rd (red arrow). (**G**) Findings for guinea pig #21 at 3 weeks: level four indicates complete loss of the blood flow signal.

As we did not have a reference for the imaging and pathology, OCTA blood flow signal patterns within the ONH regions were categorized into four levels based on our clinical experience. Level one (normal blood flow signals) showed central peak blood signals and intact peripheral vascular rings. Level two (< 1/3rd blood flow signal defect) showed a peripheral vascular ring defect of < 1/3rd but with a central peak blood signal. Level three (< 2/3rd blood flow signal defect) showed peripheral vascular ring defects of < 2/3rd or loss of central peak blood signal. Level four (> 2/3rd blood flow signal defects) had more severe vascular defects than the other three levels defined above (Fig. 1). These four levels were used to assess the severity of ONH ischemia. We aim for this classification system to benefit further studies on guinea pigs.

The guinea pig eyes were excluded from the analysis of ONH ischemia if the OCTA was of poor imaging quality or did not show normal blood flow signals. All measurements and blood flow signal patterns were assessed by two investigators. The average values of all measurements were calculated. For disagreements, the senior investigator made a final decision.

### Statistical analyses

Data are presented as the mean ± standard deviation. SPSS software (version 22.0; SPSS, Inc., Chicago, IL, USA) was used for statistical analyses. Differences in refraction, IOP, AL, and OCT-based optic disc parameters between baseline and 3 weeks were compared using the paired-sample *t*-test. Pearson correlation analysis was used to investigate the relationship between age and all the other measurement parameters, along with changes in AL and refractive error between baseline and 3 weeks. The factors influencing changes in IOP were evaluated using the Pearson correlation and linear regression model to establish the corresponding equation; 95% confidence intervals (CI) were also calculated. The factors influencing the severity of the blood signal defects were analyzed using Pearson’s correlation. A value of p < 0.05 was considered statistically significant.

## Results

**Figure 2:**
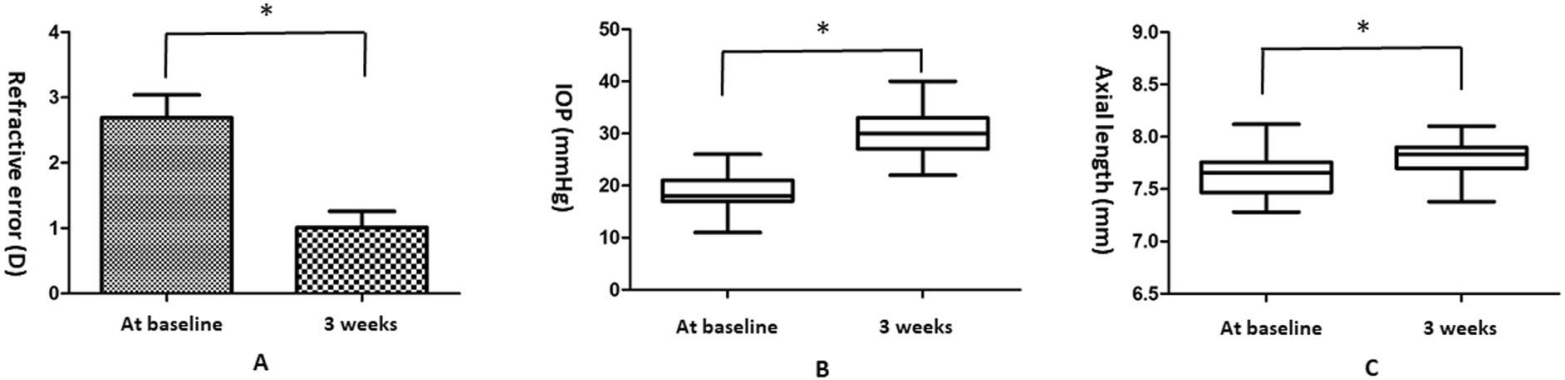
General parameters of the 88 guinea pigs. (**A**) Refractive error decreased significantly from 2.69 ± 3.235 D at baseline to 1.01 ± 2.325 D at 3 weeks (*p < 0.001). (**B**) IOP increased significantly from 18.85 ± 3.254 mmHg at baseline to 29.88 ± 3.999 mmHg at 3 weeks (*p < 0.001). (**C**) Axial length increased significantly from 7.62 ± 0.179 mm at baseline to 7.81 ± 0.146 mm at 3 weeks (*p < 0.001).

The 88 guinea pigs analyzed in this study were aged 3 (n = 29), 4 (n = 51), and 5 (n = 8) weeks. The guinea pigs’ starting ages did not affect the measured parameters (all p > 0.05). The refractive error decreased significantly from 2.69 ± 3.235 D at baseline to 1.01 ± 2.325 D at 3 weeks (*t* = 9.680, p < 0.001). The IOP increased significantly from 18.85 ± 3.254 mmHg at baseline to 29.88 ± 3.999 mmHg at 3 weeks (*t* = 24.804, p < 0.001). The AL increased significantly from 7.62 ± 0.179 mm at baseline to 7.81 ± 0.146 mm at 3 weeks (*t* = 14.071, p < 0.001; Table 1 and Fig. 2). The mean decrement of refractive error at 3 weeks was − 1.68 ± 1.630 D, which correlated negatively with the increment in AL (0.18 ± 0.123 mm, r =  − 0.784, p < 0.001).

**Table 1:** Changes in all parameters between baseline and 3 weeks (n = 88).

Parameters	Baseline (0)	3 weeks (3)	Significance	Change (3-0)
Refractive error (diopter)	2.69 ± 3.235	1.01 ± 2.325	*t* = 9.680, p < 0.001	− 1.68 ± 1.630
IOP (mmHg)	18.85 ± 3.254	29.88 ± 3.999	*t* = 24.804, p < 0.001	11.02 ± 4.169
AL (mm)	7.62 ± 0.179	7.81 ± 0.146	*t* = 14.071, p < 0.001	0.18 ± 0.123
Average C/D	0.38 ± 0.118	0.62 ± 0.0690	*t* = 23.752, p < 0.001	0.24 ± 0.0940
Vertical C/D	0.39 ± 0.130	0.62 ± 0.0669	*t* = 19.807, p < 0.001	0.23 ± 0.110
cpRNFL thickness (µm)	53.18 ± 13.894	64.86 ± 24.446	*t* = 4.893, p < 0.001	11.68 ± 22.397
Rim area (mm^2^)	0.73 ± 0.112	0.55 ± 0.0955	*t* = 16.625, p < 0.001	− 0.18 ± 0.103
Disc area (mm^2^)	0.88 ± 0.106	0.95 ± 0.151	*t* = 5.866, p < 0.001	0.074 ± 0.118
Cup volume (mm^3^)	0.028 ± 0.0159	0.090 ± 0.111	*t* = 5.193, p < 0.001	0.062 ± 0.112

IOP, intraocular pressure; AL, axial length; C/D, cup-to-disc ratio; cpRNFL, circumpapillary retinal nerve fiber layer.

**Figure 3:**
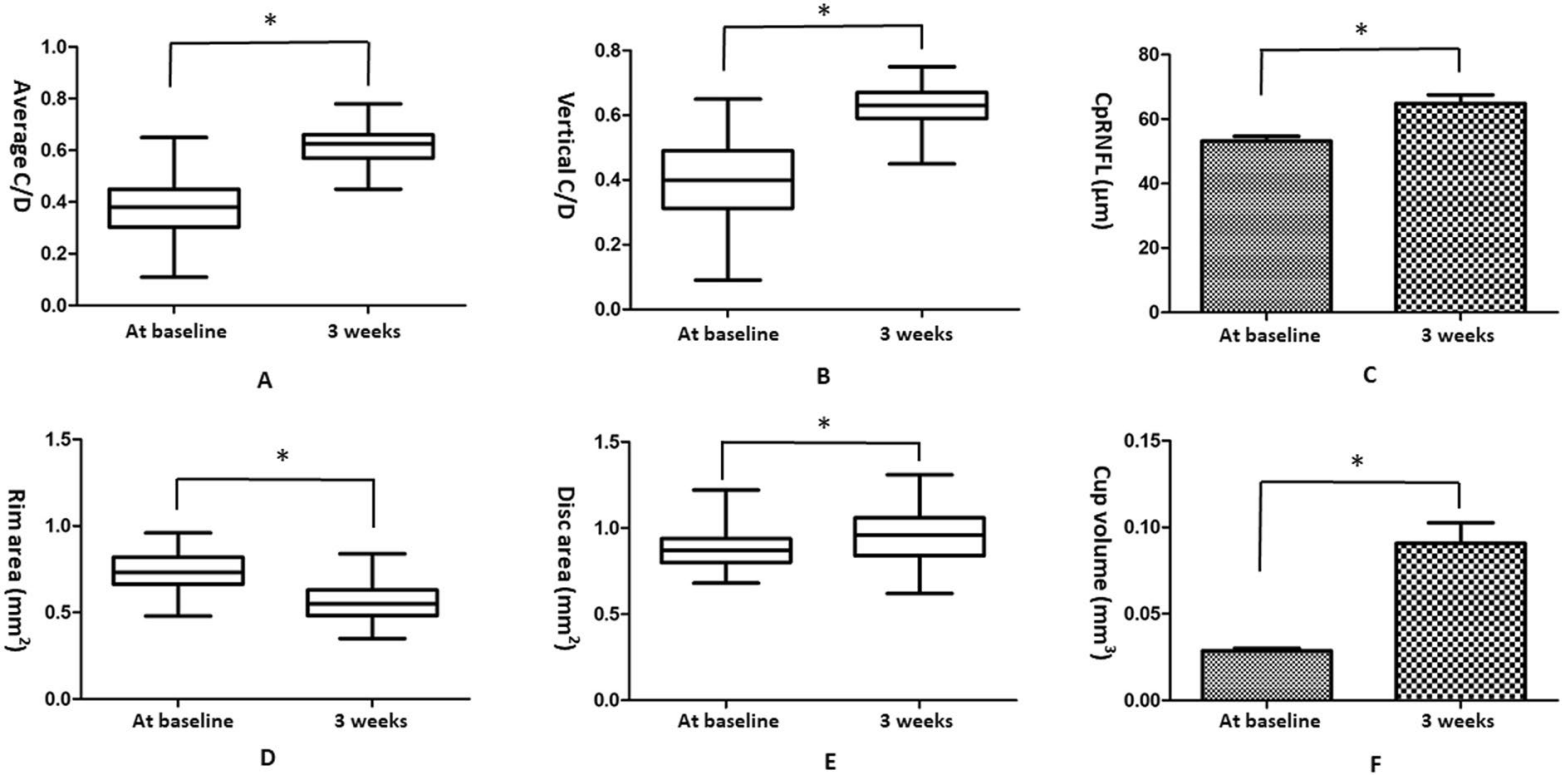
Morphological parameters of the optic nerve head in all 88 guinea pigs. (**A**) Average C/D increased significantly from 0.38 ± 0.118 at baseline to 0.62 ± 0.0690 at 3 weeks (*p < 0.001). (**B**) Vertical C/D increased significantly from 0.39 ± 0.130 at baseline to 0.62 ± 0.0669 at 3 weeks (*p < 0.001). (**C**) cpRNFL thickness increased significantly from 53.18 ± 13.894 µm at baseline to 64.86 ± 24.446 µm at 3 weeks (*p < 0.001). (**D**) Rim area decreased significantly from 0.73 ± 0.112 mm^2^ at baseline to 0.55 ± 0.0955 mm^2^ at 3 weeks (*p < 0.001). (**E**) Disc area increased significantly from 0.88 ± 0.106 mm^2^ at baseline to 0.95 ± 0.151 mm^2^ at 3 weeks (*p < 0.001). (**F**) Cup volume increased significantly from 0.028 ± 0.0159 mm^3^ at baseline to 0.090 ± 0.111 mm^3^ at 3 weeks (*p < 0.001). C/D, cup-to-disc ratio; cpRNFL, circumpapillary retinal nerve fiber layer. Data are shown as mean ± standard deviation.

The average C/D ratio increased significantly from 0.38 ± 0.118 at baseline to 0.62 ± 0.0690 at 3 weeks (*t* = 23.752, p < 0.001). The vertical C/D ratio increased significantly from 0.39 ± 0.130 at baseline to 0.62 ± 0.0669 at 3 weeks (*t* = 19.807, p < 0.001). The cpRNFL thickness increased significantly from 53.18 ± 13.894 µm at baseline to 64.86 ± 24.446 µm at 3 weeks (*t* = 4.893, p < 0.001). The rim area decreased significantly from 0.73 ± 0.112 mm^2^ at baseline to 0.55 ± 0.0955 mm^2^ at 3 weeks (*t* = 16.625, p < 0.001). The disc area increased significantly from 0.88 ± 0.106 mm^2^ at baseline to 0.95 ± 0.151 mm^2^ at 3 weeks (*t* = 5.866, p < 0.001). The cup volume increased significantly from 0.028 ± 0.0159 mm^3^ at baseline to 0.090 ± 0.111 mm^3^ at 3 weeks (*t* = 5.193, p < 0.001; Table 1 and Fig. 3).

The increase in IOP showed a positive relationship with the increase in average C/D (r = 0.581, p < 0.001), vertical C/D (r = 0.473, p < 0.001), and cup volume (r = 0.232, p = 0.030); and a negative relationship with increment in the rim area (r =  − 0.555, p < 0.001). IOP was related positively to the average C/D (B = 16.204, *t* = 2.103, p = 0.039, 95% CI: 0.876–31.533) and negatively to the rim area (B =  − 12.149, *t* = 2.689, p = 0.009, 95% CI: − 21.138 to 3.160). Linear regression indicated the equation for calculating IOP change = 16.204 × (change in average C/D) − 12.149 × (change in rim area) + 3.752 (Table 2).

**Table 2:** Factors influencing the increment in IOP.

Influencing parameters	B	β	*t*	p	95% CI
Constant	3.752		1.503	0.137	− 1.214 to 8.718
Increment in average C/D	16.204	0.365	2.103	0.039	0.876 to 31.533
Increment in vertical C/D	0.271	0.007	0.047	0.963	− 11.198 to 1.739
Increment in rim area	− 12.149	− 0.299	2.689	0.009	− 21.138 to 3.160
Increment in cup volume	5.953	0.159	1.850	0.068	− 0.448 to 12.355
Age	0.206	0.030	0.344	0.732	− 986 to 1.398

IOP, intraocular pressure; B, regression coefficient; β, standardized regression coefficient; t, statistical value; p, statistical significance; CI, confidence interval; C/D, cup-to-disc ratio.

The 42 of 88 guinea pigs that underwent OCTA examinations of the ONH were aged 3 (n = 17), 4 (n = 19), and 5 (n = 6) weeks. At baseline, the eyes of 42 guinea pigs showed normal blood flow signals in the ONH region. At 3 weeks, the eyes showed level 1 (n = 3), 2 (n = 11), 3 (n = 16), and 4 (n = 12) findings. The severity of the blood signal defects (ONH ischemia) showed a positive relationship with the average C/D at 3 weeks (r = 0.342, p = 0.027).

## Discussion

To the best of our knowledge, this study is the first to use OCT to study the relationship between morphological and vascular glaucomatous changes driven by increased IOP due to FD and CXL in guinea pigs. We also characterized four levels of ONH blood flow signal patterns using OCTA and analyzed their relationships with morphological parameters. The ONH parameters in normal guinea pigs were previously quantified using both OCT and OCTA; the analyses found no significant differences in the total number of junctions and endpoints, total vessel length, average vessel area, and vascular density and diameter within the ONH region between 3- and 4-week-old normal guinea pigs^9^. This study is a follow-up of previous research under high IOP conditions.

Three weeks after the FD + CXL procedure, the IOP and AL increased, along with a corresponding reduction in the refractive error. This finding is consistent with the results of Wang et al., who reported that scleral CXL led to less myopia and axial elongation; however, the procedure yielded outcomes comparable to those in untreated control guinea pigs^12^. In addition, Mattson et al. found that IOP increased during intact globe expansion in the eyes of rabbits with CXL^13^. Moreover, a previous study showed that FD + CXL elevated IOP compared to FD or CXL alone. This increase in IOP was time-dependent because of CXL-related scleral stiffness and post-scleral vascular resistance^8^ that was related to myopia development, high IOP, and glaucoma^14^.

At baseline, the cpRNFL thickness, average and vertical C/D, disc area, rim area, and cup volume were 53.18 ± 13.894 µm, 0.38 ± 0.118 and 0.39 ± 0.130, 0.88 ± 0.106 mm^2^, 0.73 ± 0.112 mm^2^, and 0.028 ± 0.0159 mm^3^, respectively, consistent with previous data^9^. In addition to increased IOP, glaucomatous changes in the ONH were observed quantitatively. The average and vertical C/D (0.24 ± 0.0940 and 0.23 ± 0.110), cpRNFL thickness (11.68 ± 22.397 µm), disc area (0.074 ± 0.118 mm^2^), and cup volume (0.062 ± 0.112 mm^3^) at 3 weeks after the FD + CXL procedure increased significantly compared to baseline values. The rim area of the ONH significantly decreased (− 0.18 ± 0.103 mm^2^).

Similarly, Kimball et al.^15^ found that scleral cross-linking by glyceraldehyde treatment in eyes showed greater retinal ganglion cell axon loss due to elevated IOP compared with either buffer-injected or control eyes. They reported experimental alterations in the sclera by crosslinking and increased susceptibility to retinal ganglion cell damage in mice^15^. The present study’s findings are consistent with clinical reports of human glaucoma^16,17^. For the first time, we summarized a relationship equation quantitatively describing structural changes in the ONH and IOP. The equation provides a reference for further glaucomatous and myopic research based on the guinea pig model.

In normal guinea pigs, the ONH total vessel length is positively affected by cpRNFL thickness and negatively by IOP^8^. This study categorized the vascular patterns of the ONH into four levels to analyse the severity of ONH ischemia. The analyses suggested that the severity of the blood signal defects had a positive relationship with the average C/D ratio at 3 weeks. Rao et al. also reported that lower whole-enface disc vessel densities were significantly associated with a faster decline in mean deviation^18^. The starting age of CXL showed no significant relationship with all parameters; thus, the results would not be influenced by age (3–5 weeks of age).

This study has limitations. First, OCTA images were not acquired from the eyes of some guinea pigs because of poor imaging quality. Second, a control group without FD + CXL was not enrolled. Finally, the repeatability of the OCT measurements was not analysed statistically, which may lead to the unreliability of our results. We will address these limitations in future research.

In conclusion, this study is the first to describe the vascular characteristics of the ONH using OCTA; the results are consistent with the morphological changes. Guinea pigs show glaucomatous changes caused by increased IOP induced by FD and CXL. Further research should focus on glaucomatous damage in myopic guinea pig models.

## Data Availability

The datasets used and/or analysed during the current study are available from the corresponding author upon reasonable request.

## Abbreviations

ONH: Optic nerve head
LC: Lamina cribrosa
OCT: Optical coherence tomography
OCTA: OCT angiography
IOP: High intraocular pressure
AL: Axial length
FD: Form deprivation
CXL: Scleral crosslinking
cpRNFL: Circumpapillary retinal nerve fibre layer
C/D: Cup-to-disc
CI: Confidence intervals
