# Publisher Correction: Morphological and vascular evidence of glaucomatous damage in myopic guinea pigs with scleral crosslinking

**DOI:** 10.1038/s41598-024-51691-7

**Published:** 2024-01-10

**Authors:** Lei Guo, Jun Tao, Ziqi Guo, Yang Tong, Shichao Chen, Xin Zhao, Rui Hua

**Affiliations:** 1 Ophthalmology and Optometry Centre, The First Hospital of China Medical University, Shenyang, China; 2 Department of Ophthalmology, Shenyang Sinqi Eye Hospital, Shenyang, China; 3 The First Clinical College of Jinzhou Medical University, Jinzhou, China; 4 Department of Ophthalmology, The First Hospital of China Medical University, No. 155, Nanjingbei Street, Heping District, Shenyang, 110001 Liaoning People’s Republic of China

Correction to: *Scientific Reports* 10.1038/s41598-023-48461-2, published online 02 January 2024

In the original version of this Article, the author Ziqi Guo was incorrectly listed as a corresponding author.

The original Article has been corrected.

